# Silencing *COI1* in Rice Increases Susceptibility to Chewing Insects and Impairs Inducible Defense

**DOI:** 10.1371/journal.pone.0036214

**Published:** 2012-04-27

**Authors:** Mao Ye, Shi Ming Luo, Jie Fen Xie, Yan Fang Li, Tao Xu, Yang Liu, Yuan Yuan Song, Keyan Zhu-Salzman, Ren Sen Zeng

**Affiliations:** 1 State Key Laboratory of Conservation and Utilization of Subtropical Agro-bioresources, Ministry of Agriculture, South China Agricultural University, Wushan, Guangzhou, People's Republic of China; 2 Key Laboratory of Tropical Agro-environment, Ministry of Agriculture, South China Agricultural University, Wushan, Guangzhou, People's Republic of China; 3 Plant Protection Institute, Guangdong Academy of Agricultural Sciences of China, Guangzhou, People's Republic of China; 4 College of Life Sciences, South China Agricultural University, Wushan, Guangzhou, People's Republic of China; 5 Department of Entomology, Texas A&M University, College Station, Texas, United States of America; Max Planck Institute for Chemical Ecology, Germany

## Abstract

The jasmonic acid (JA) pathway plays a key role in plant defense responses against herbivorous insects. CORONATINE INSENSITIVE1 (COI1) is an F-box protein essential for all jasmonate responses. However, the precise defense function of COI1 in monocotyledonous plants, especially in rice (*Oryza sativa* L.) is largely unknown. We silenced *OsCOI1* in rice plants via RNA interference (RNAi) to determine the role of *OsCOI1* in rice defense against rice leaf folder (LF) *Cnaphalocrocis medinalis*, a chewing insect, and brown planthopper (BPH) *Nilaparvata lugens*, a phloem-feeding insect. In wild-type rice plants (WT), the transcripts of *OsCOI1* were strongly and continuously up-regulated by LF infestation and methyl jasmonate (MeJA) treatment, but not by BPH infestation. The abundance of trypsin protease inhibitor (TrypPI), and the enzymatic activities of polyphenol oxidase (PPO) and peroxidase (POD) were enhanced in response to both LF and BPH infestation, but the activity of lipoxygenase (LOX) was only induced by LF. The RNAi lines with repressed expression of *OsCOI1* showed reduced resistance against LF, but no change against BPH. Silencing *OsCOI1* did not alter LF-induced LOX activity and JA content, but it led to a reduction in the TrypPI content, POD and PPO activity by 62.3%, 48.5% and 27.2%, respectively. In addition, MeJA-induced TrypPI and POD activity were reduced by 57.2% and 48.2% in *OsCOI1* RNAi plants. These results suggest that *OsCOI1* is an indispensable signaling component, controlling JA-regulated defense against chewing insect (LF) in rice plants, and COI1 is also required for induction of TrypPI, POD and PPO in rice defense response to LF infestation.

## Introduction

Plants are frequently exposed to herbivorous insect attack and microbial pathogen infection in the natural environment. Different defense mechanisms are activated in response to potential enemies via several interacting signaling pathways, including the jasmonate (JA), salicylate (SA) and ethylene (ET) pathways. Jasmonates (JAs) are derived from linolenic acid and characterized by a pentacyclic ring structure [1], [2]. The jasmonate pathway plays a key role in plant defense responses against herbivorous insects. In many plant species, insect feeding activates a wide variety of genes that are responsive to JA and related octadecanoids, including methyl jasmonate (MeJA) and 12-oxo-phytodienoic acid (OPDA) [3]. It has been well studied that feeding damage by herbivorous insect elicits a rapid burst of octadecanoid signals in dicotyledonous plants, such as *Arabidopsis thaliana*, tobacco (*Nicotiana attenuata*), and tomato (*Lycopersicon esculentum*) [4]–[6], to trigger production of defense compounds and anti-nutritive substances that deter further insect damage [7]–[11]. The jasmonate pathway also regulates production of volatiles in tomato plants, which can attract natural enemies of herbivorous insects [12].

In dicotyledonous plants, mutants impaired in JA biosynthesis and perception have been examined for effects on plant-herbivore interactions. Coronatine, a phytotoxin produced by the plant pathogen *Pseudomonas syringae*, acts as a molecular mimic of jasmonoyl-isoleucine (JA-Ile) and activates JA signaling [13]–[16]. CORONATINE INSENSITIVE1 (COI1) is an F-box protein and has been implicated in jasmonate-regulated defense responses [17]. COI1 interacts with multiple proteins to form the SCF^COI1^ E3 ubiquitin ligase complex and recruits JASMONATE ZIM-DOMAIN (JAZ) proteins for degradation by the 26S proteasome. The physical interaction of COI1 with the JAZ protein is promoted by an Ile-conjugated form of jasmonic acid (JA-Ile) to serve as a receptor for jasmonate and activate the JA signaling pathway [18]–[21]. COI1 is required for expression of approximately 84% of 212 JA-induced genes in *Arabidopsis* [22].

Our current understanding of JA function in dicotyledonous plants mainly derives from analyses of mutants with alteration in either JA biosynthesis or signal transduction. Recently, mutants defective in the perception of JA including *coi1*, *jar1*, *jin1*, and *jin4* have been widely used in study of JA signaling [17], [23], [24]. Of these characterized JA-insensitive mutants, *coi1* is the least responsive to JA and has been used extensively to study the effects of JA signaling in various plant processes. The *coi1* mutant is male-infertile, and insensitive to JA-mediated root growth inhibition [25]–[27]. Likewise, *coi1* mutants are more sensitive to insects in *Arabidopsis*, tobacco and tomato plants [11], [28], [29], [30]. For example, *COI1*-silenced tobacco plants do not activate nicotine biosynthesis genes after jasmonate treatment or wounding on leaves, which lead to reduced resistance against larvae of *Manduca sexta* [31]. The two-spotted spider mite (*Tetranychus urticae*) preferred the tomato *coi1* mutant over WT plants in choice assays, and laid more eggs on the mutant plants [15]. Recent studies have found that COI1 involves inositol polyphosphates [32] and ethylene-induced root growth inhibition in the light in *Arabidopsis thaliana* [33]. In *Solanum nigrum*, COI1 controls jasmonate metabolism and the production of a systemic signal against insect attack [34].

Interestingly, plants activate different signaling pathways in response to different insect feeding styles, leading to the production of different defensive compounds [35]–[37]. In general, chewing herbivorous insects induce JA-regulated defense [38], [39], whereas piercing-sucking insects tend to trigger expression of genes and the synthesis of defense compounds similar to those activated by fungal or bacterial pathogens [40]–[45]. Aphid feeding, for instance, induces the transcription of genes regulated by SA signaling pathways [46]–[48].

Relative to dicots, COI1-mediated resistance to herbivorous insects in monocots is largely unknown. Hu *et al.* [49] firstly isolated a putative *OsCOI1* gene (accession: AY168645) from rice with 74% sequence identity to *COI1* gene in *Arabidopsis*, and its expression has been confirmed to be regulated by JA. Later, Mukesh *et al.* [50] identified 687 potential F-box proteins from rice and classified them into 10 subfamilies based on their domain composition. Two F-box proteins (Os05g37690, Os01g63420) represent the closely related orthologs of *Arabidopsis* COI1 and thus may perform similar functions in rice. These two genes show 65% and 100% sequence identity to the gene isolated by Hu *et al.* [49]. Mei *et al.* [51] have successfully silenced *OsCOI1* gene in rice plants by using RNA interference technology. However, the function of COI1 in rice plants remains unknown.

In the present study, to elucidate the role of *OsCOI1* in insect-induced defense responses in rice plants, we silenced the gene *OsCOI1* (accession: AY168645) isolated by Hu et al. [49] via RNA interference technology. The relative expression levels of defense related genes, activities of defense-related enzymes (PPO, POD, LOX), production of TrypPI, JA and SA levels were compared between *OsCOI1* RNAi lines and wild-type plants (WT) in response to brown planthopper (BPH) *Nilaparvata lugens*, a phloem-feeding insect, and rice leaf folder (LF) *Cnaphalocrocis medinalis*, a chewing insect. We also examined the differential performance of the two insects on WT and *OsCOI1* RNAi plants.

## Results

### *OsCOI1* transcripts induced by insect infestation and MeJA treatment in WT plants

**Figure 1:**
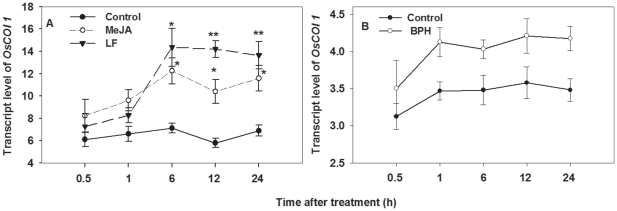
Transcript level of *OsCOI1* in wild-type (WT) rice plants. (**A**) WT plants treated with methyl jasmonate (MeJA) and rice leaf folder (LF), (**B**) WT plants treated with brown planthopper (BPH). qRT-PCR was used to detect the transcript levels. Values are mean ± standard error of three biological replicates. For each time point, asterisks indicate significant difference in treated plants compared to untreated control plants respectively ( *P<0.05, **P<0.01 according to Student*'*s *t*-test).

To determine transcript response of *OsCOI1* to insect infestation and exogenous MeJA application in WT rice plants, we performed a time-course real-time PCR analysis. Leaf tissue (or leaf sheath tissue) was harvested from individual plants at different time points after infestation by LF (or BPH) or application of 1 mM MeJA. *OsCOI1*-specific qRT-PCR revealed that *OsCOI1* transcripts were up-regulated by MeJA and LF infestations. *OsCOI1* transcripts accumulated to 1.88-, 2.41- and 1.98-fold higher levels in response to LF infestation at 6, 12 and 24 h, respectively (F_1, 29_ = 17.8, P<0.01) (Fig. 1A). *OsCOI1* transcripts were induced approximately 1.99-, 2.04- and 1.68-fold by MeJA treatment at 6, 12 h and 24 h, respectively (F_1, 29_ = 34.04, P<0.01). However, BPH infestation did not significantly change the transcript abundance of *OsCOI1* (F_1, 29_ = 0.951, P = 0.338) (Fig. 1B). These results suggest that *OsCOI1* may only be involved in JA-related rice defense to chewing insects.

### Differential induction of TrypPI level and enzymatic activities by LF and BPH

**Figure 2:**
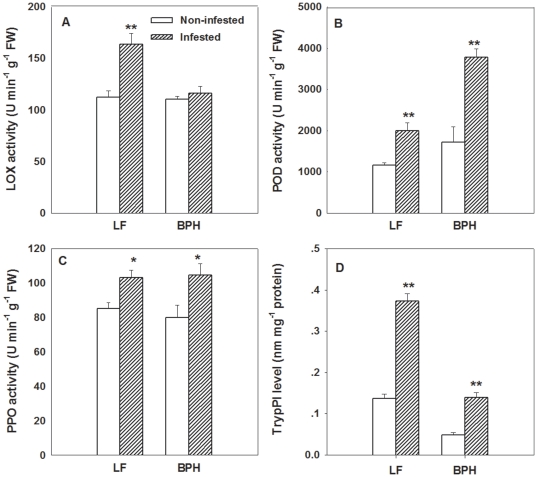
Levels of three defence-related enzymes and TrypPI in leaves of wild-type (WT) rice plants infested with BPH and LF. Three defence-related enzymes included: (**A**) lipoxygenase (LOX), (**B**) peroxidase (POD), (**C**) polyphenol oxidase (PPO). Values are mean ± standard error of six biological replicates. Asterisks indicate significant differences of herbivore infested plants compared to control non-infested plants (*P<0.05, **P<0.01 according to Student*'*s *t*-test).

Enzymatic activity analyses revealed that activity of lipoxygenase (LOX), which catalyzes the initial reaction in JA biosynthesis pathway [52], increased by 48.7% in LF-infested plants compared with non-infested WT plants, while BPH feeding did not change LOX activity (Fig. 2A). Activities of polyphenol oxidase (PPO), which oxidizes phenolics to highly toxic quinones [53], and peroxidase (POD), which catalyzes the formation of lignin and other oxidative phenols to prevent insect consumption [54], were enhanced by 21.3% and 72.3%, respectively in response to LF feeding in WT plants. Likewise, BPH infestation increased activities of PPO and POD by 30.6% and 119.1%, respectively (Fig. 2B, C). These results showed that PPO and POD are induced by both LF and BPH infestation, while LOX is only induced by LF.

Protease inhibitors (PIs) have been implicated in plant defense against lepidopteran herbivorous insects via interfering with their digestive process [55], [56]. In our study, we found that trypsin protease inhibitor (TrypPI) levels in WT plants were induced by 107% and 130% by BPH and LF infestation (respectively), compared with un-infested control plants (Fig. 2D).

### Silencing *OsCOI1* reduces rice resistance to LF but not to BPH

Southern blot analysis showed that a single copy of the *OsCOI1* RNAi construct was inserted into the genome of *Oryza sativa* L. in RNAi lines (Fig. S4A). In addition, RT-PCR analysis showed that *OsCOI1* expression was significantly down-regulated in RNAi lines, and could not be recovered by JA treatment (Fig. S4B). The *OsCOI1* RNAi lines showed earlier and less tillering compared with WT, and most RNAi lines yielded empty grain (Fig. S5), suggesting a role of *COI1* in rice fertility. There were few seeds in each line. Therefore T_2_ seeds were pooled for functional analysis for most experiments.

**Figure 3:**
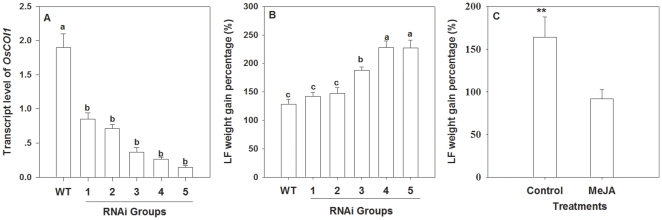
Transcript level of *OsCOI1* and LF performance in wild-type (WT) and *OsCOI1* RNAi rice plants. (**A**) Transcript level of *OsCOI1* in WT and five groups of RNAi lines. (**B**) Weight gain percentage (%) of individual LF larvae after 3 days feeding on each line (WT: wild type rice plants; 1–5: five groups of RNAi lines). (**C**) Weight gain percentage (%) of individual LF larvae after 3 days feeding on WT plants, which had been either individually sprayed with 1 ml of 1 mM MeJA with 0.01% Tween 20 (MeJA), or with 0.01% Tween 20 (untreated control) for 48 h in advance. Values are mean ± standard error of at least three biological replicates. Letters above bars indicate significant differences among WT and five groups of RNAi lines (*P<0.05* according to Tukey's multiple range test). Asterisks indicate significant differences in MeJA-treated WT plants compared to untreated control plants (*P<0.05, **P<0.01 according to Student*'*s *t*-test).

**Figure 4:**
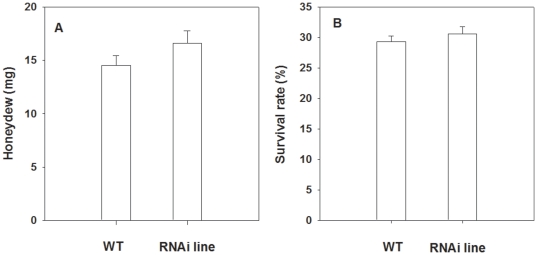
Honey dew and survival rate of BPH feeding on wild-type (WT) and *OsCOI1* RNAi rice lines. (**A**) Amount of honeydew per day secreted by three female BPH adults and (**B**) Survival rate of BPH nymphs after 5 days feeding on each line. Values are mean ± standard error of ten replicates, asterisks indicate significant differences in RNAi lines compared to WT (*P<0.05 according to Student's *t*-test).

qRT-PCR analysis revealed different expression levels of *OsCOI1* in 30 RNAi plants, According to the expression level of *OsCOI1*, these 30 plants were divided into five groups, which transcripts level were only 7.4% to 39.5% of that in WT plants (F_5, 34_ = 14.24, P<0.01) (Fig. 3A). In each group, there were six plants with approximately equal expression level of *OsCOI1*. Additionally, 15 WT plants served as control. Two second instar LF larvae were placed individually on the node 3 and 4 leaves of WT and RNAi plants. By day 3, the mean weight gain percentage (%) of LF larvae feeding on the RNAi group 3, 4 and 5 were 1.47-, 1.79- and 1.78-times of those feeding on WT plants (F_5, 89_ = 40.62, P<0.01) (Fig. 3B). Additionally, larvae on MeJA-treated WT plants got 51.7% less weight gain compared with those feeding on untreated control plants (Fig. 3C). In contrast, BPH showed no significant difference between WT and RNAi lines. The amounts of honeydew secreted per day by a BPH female adult, an indicator of the amount of food intake, did not show a significant difference between those feeding on WT and RNAi lines (Fig. 4A). Also, the survival rate of BPH nymphs feeding on WT plants had no significant difference from those feeding on RNAi lines (Fig. 4B). The obvious differences in LF weight gain between those feeding on WT and RNAi lines demonstrate the important role of *OsCOI1* in rice resistance against LF. The result that BPH showed no difference between WT and RNAi lines suggests that the reduced expression of *OsCOI1* does not negatively affect rice resistance against BPH, in agreement with the weak induction of *OsCOI1* transcript by BPH (Fig. 1B).

### JA-mediated insect-induced responses are *OsCOI1*-dependent

To determine the exact role of *OsCOI1* in response to LF, we evaluated JA and SA concentration, as well as the TrypPI content and enzymatic activities of LOX, POD, and PPO in WT plants and RNAi plants with or without LF infestation.

**Figure 5:**
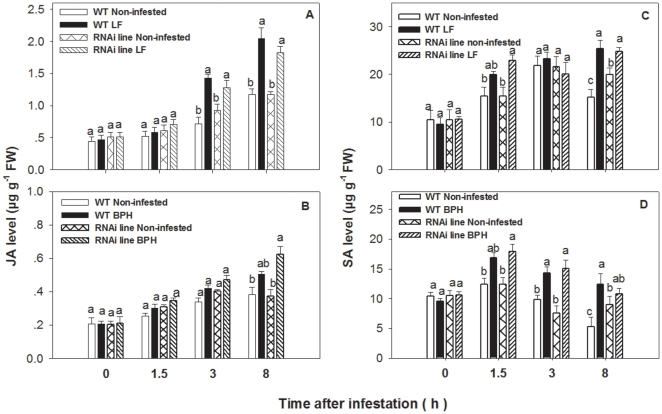
JA and SA levels in wild-type (WT) and *OsCOI1* RNAi rice plants infested with LF (A, C) and BPH (B, D). Values are mean ± standard error of six biological replicates. For each time point, letters indicate significant differences among treatments (*P<0.05* according to Tukey's multiple range test).

Results showed that JA levels were significantly higher 3 and 8 h after LF infestation in both WT plants (F_1, 47_ = 4.318, P = 0.044) and RNAi plants (F_1, 47_ = 5.582, P = 0.022) as compared to the non-infested control. JA levels in RNAi plants were not significantly lower at 3 and 8 h compared to those in WT plants (F_1, 47_ = 0.085, P = 0.967) (Fig. 5A), suggesting that LF-induced JA level was not affected by *OsCOI1* silencing. BPH infestation did not increase JA level in both WT and RNAi plants (F_1, 47_ = 1.56, P = 0.21) (Fig. 5B).

SA levels in BPH-infested WT plants were significantly higher 1.5, 3 and 8 h after insect infestation than those in the non-infested WT plants (F_1, 47_ = 45.81, P<0.01). BPH-infested RNAi plants showed the same trend (F_1, 47_ = 16.94, P<0.01) (Fig. 5D). There was no significant difference in SA levels between WT and *OsCOI1* RNAi plants 1.5, 3 and 8 h after BPH infestation (F_1, 47_ = 1.67, P = 0.23), suggesting that *OsCOI1* silencing does not change BPH-induced SA levels. LF infection also increased SA levels in WT plants 8 h after treatment (F_1, 48_ = 11.29, P<0.01). Silencing *OsCOI1* in rice did not reduce LF-induced SA levels (F_1, 47_ = 24.59, P<0.01) (Fig. 5C).

**Figure 6:**
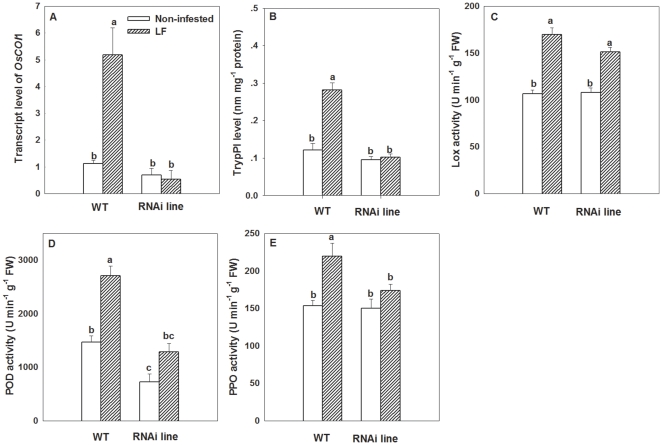
Levels of *OsCOI1* transcripts (A), TrypPI (B) and three defence-related enzymes in wild-type (WT) and *OsCOI1* RNAi rice plants infested with LF. Three defence-related enzymes included: (**C**) lipoxygenase (LOX), (**D**) peroxidase (POD), (**E**) polyphenol oxidase (PPO). qRT-PCR was used to detect the transcript levels. Values are mean ± standard error of six biological replicates. Letters above bars indicate significant differences among four treatments (*P<0.05* according to Tukey's multiple range test).

LF infestation strongly induced the transcripts of *OsCOI1* (F_3, 11_ = 19.44, P<0.01) and enhanced TrypPI level in WT plants (Fig. 6A). However, *OsCOI1* silencing impaired the inducibility of *OsCOI1* transcripts and TrypPI by LF, and there was no significant change in *OsCOI1* transcripts and TrypPI level in RNAi lines after LF feeding (Fig. 6A). The TrypPI level in RNAi lines was only 38.7% of that in infested WT plants (F_3, 23_ = 30.77, P<0.01) (Fig. 6B).

There was no significant difference in LOX activity between WT plants and RNAi plants with LF infestation (Fig. 6C), indicating that silencing *OsCOI1* did not change LOX activity. However, the suppressed expression of *OsCOI1* resulted in 50.9% reduction in POD activity compared to that in non-infested WT plants. LF-induced POD activity was significantly reduced (by 48.5%) in *OsCOI1* RNAi plants compared to that in LF-infested WT plants (F_3, 23_ = 28.65, P<0.01) (Fig. 6D). PPO activity in RNAi plants did not differ significantly from that in WT plants without LF infestation, but PPO activity in LF-infested RNAi plants was significantly reduced by 27.2% compared to that in LF-infested WT plants (F_3, 23_ = 10.26, P<0.01) (Fig. 6E). These results suggest that *OsCOI1* is required for the induction of POD, PPO and TrypPI activities in the rice defense response to LF.

**Figure 7:**
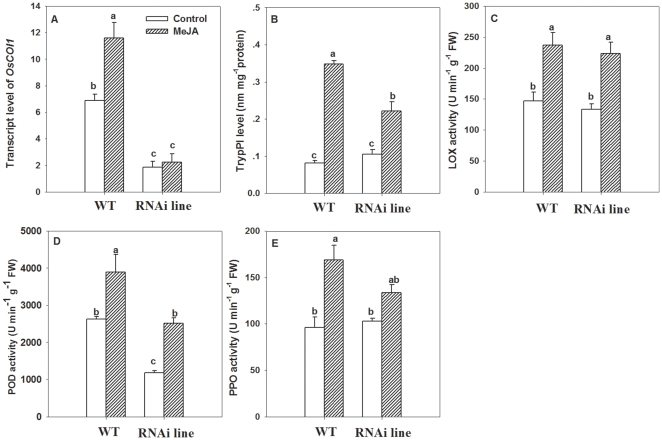
Levels of *OsCOI1* transcripts (A), TrypPI (B) and three defence-related enzymes in wild-type (WT) and *OsCOI1* RNAi rice plants treated with MeJA. Three defence-related enzymes included: (**C**) lipoxygenase (LOX), (**D**) peroxidase (POD), (**E**) polyphenol oxidase (PPO). qRT-PCR was used to detect the transcript levels. Values are mean ± standard error of six biological replicates. Letters above bars indicate significant differences among four treatments (*P<0.05* according to Tukey's multiple range test).

Additionally, transcripts of *OsCOI1* in WT plants were induced by 89.2% with exogenous MeJA application (Figure 7A). TrypPI, LOX, POD and PPO activities were enhanced by MeJA by 322.2%, 54.6%, 42.9% and 71.6% respectively (Figure 7B–E). However, MeJA-induced TrypPI and POD activities were reduced by 57.2% and 48.2% in RNAi plants compared to those of MeJA-treated WT plants (Figure 7B, D). These results demonstrate that *OsCOI1* is required for MeJA-induced rice defense, including POD and TrpyPI activities.

## Discussion

In this study, a comparison of resistance against chewing and phloem-feeding insects between wild-type (WT) and *OsCOI1* silenced RNAi plants provides new insight into the role of *COI1* in rice defense against herbivorous insects. We found that LF infestation and MeJA application strongly and constantly enhanced the transcript levels of *OsCOI1* in WT plants (Fig. 1A), but BPH only slightly induced *OsCOI1* transcripts (Fig. 1B). Lipoxygenase enzyme (LOX), a key component in JA biosynthesis [52], was significantly induced by LF but not by BPH (Fig. 2A). In addition, LF induced higher levels of JA production in WT plants (Fig. 5A), while BPH infestation significantly increased SA in WT plants (Fig. 5D). These results suggested that COI1 and the JA signaling pathway are involved in rice resistance against LF but not BPH.

Insect infestation elicits a burst of JA signaling in plants [10], [36], resulting in JA accumulation [18], [22], [48]. Our study showed that silencing *OsCOI1* did not reduce LF-induced LOX activity (Fig. 6C) and JA levels (Fig. 5A), nor did it reduce MeJA-induced LOX activity (Fig. 7C), suggesting that *OsCOI1* is not involved in JA biosynthesis in rice plants. It is likely that COI1 acts as a receptor in the JA signal pathway in monocots, as its counterparts in dicots [18], [33], [57].

Increases in activities of PPO [53], POD [12], [54], and TrypPI [55], [56] are the most prominent systemic responses against insect feeding in plants. All of these proteins have been demonstrated to reduce the nutritive value of plant foliage to herbivorous insects. Our study confirmed that POD, PPO activities and TrypPI production in rice were all increased in response to both LF and BPH infestation (Fig. 2B–D), implicating their roles in rice resistance to LF and BPH.

Silencing *OsCOI1* in rice led to improved performance of the chewing insect LF (Fig. 3A and B). Meanwhile, it decreased LF-induced TrypPI levels (Fig. 6B), POD and PPO enzymatic activities (Fig. 6D, E), demonstrating that the *OsCOI1* plays a crucial role in rice defense against LF, and that *OsCOI1* is required for induction of POD, PPO and TrypPI in rice responses to LF. Indeed, POD, PPO and TrypPI have been demonstrated to be involved in rice defense responses to LF [58], [59]. Hence the reduction of herbivore resistance in *OsCOI1* RNAi plants may be partially caused by decreased induction of POD, PPO and TrypPI. Likewise, the enhanced rice resistance to LF (Fig. 3C) by MeJA application can partially be explained by an increase in MeJA-induced TrypPI, POD, and PPO activities (Fig. 7B, D, E). The weakened induction of POD and TrypPI activities in *OsCOI1* RNAi plants by MeJA treatment (Fig. 7B, D and E) indicates that COI1 is a key regulator of MeJA-induced defense [22], [31].

Plants have evolved complex strategies to protect themselves against pests. Phloem-feeding insects tend to induce SA-mediated resistance as pathogens do [46], [47]. BPH infestation induces *PAL* and *NPR1* genes, which are the key regulators of SA-dependent systemic acquired resistance. Likewise, some PR genes regulated by the SA pathway are induced by BPH [60], [61]. Our results reveal that BPH infestation induced higher levels of SA (Fig. 5D) but not JA (Fig. 5B). Silencing *OsCOI1* did not alter the amount of honeydew (Fig. 4A) or survival rate (Fig. 4B) of BPH, implying that rice resistance to BPH, a homopteran phloem feeder of rice, is *OsCOI1*-independent.

It is generally assumed that JA and SA signaling pathways are mutually antagonistic in plant defense [62], [63]. Impaired JA signaling by suppressed expression of *OsHI-LOX* leads to increased SA-dependent resistance to BPH [59]. However, in this study silencing *OsCOI1* did not increase BPH-induced SA levels (Fig. 5D), nor it increased rice resistance to BPH (Fig. 4A, B). No antagonistic interaction between the two signaling pathways was found. The possible reason could be that silencing *OsCOI1* did not affect JA accumulation, and thereby the antagonism did not occur.

In addition to the role in herbivore resistance, *COI1* plays a central role in fertility. In tomato plants, silencing COI1 results in defective maternal control of seed maturation, as well as altered the trichome shape and number [15]. In *Nattenuate* and *Arabidopsis*, the sterility is mainly caused by defective dehiscence [17] and shorter stamens in flowers [30]. It appears that the suppressed expression of COI1 leads to different flower phenotypes in dicotyledonous plants. In rice, silencing *OsCOI1* resulted in earlier and less tillering compared to WT plants. Most *OsCOI1*-deficient plants yielded empty grain (Fig. S5), suggesting that COI1 is essential for development of fertile flowers and viable seeds in rice, although its mechanism remains to be examined.

**Figure 8:**
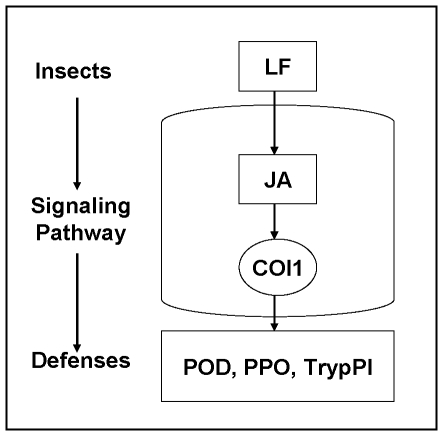
Schematic summary of the crucial role of *OsCOI1* in JA-regulated defence responses to chewing insect (LF, rice leaf folder).

Based on the results that *OsCOI1* is responsive to LF infestation and MeJA, and that *COI1* silencing in rice increases susceptibility to chewing insects and impairs the inducibility of TrypPI, PPO and POD, we conclude that the JA signal transduction pathway plays a key role in rice defense against chewing insects, and COI1 is specifically required for the regulation of JA-mediated insect defense in response to the chewing insect LF, but not for SA-mediated defense in response to BPH. Moreover, TrypPI, POD, PPO and LOX are JA-induced defense responses to the chewing insect LF. TrypPI, POD and PPO are all *OsCOI1*-mediated (Fig. 8). We therefore propose that rice plants can recognize different signals induced by chewing insects and phloem-feeding insects. In response to chewing insects, rice plants activate the JA signaling pathway leading to increases in LOX activity, increased JA level, and up regulation of *OsCOI1*. *OsCOI1* serves as a receptor of the JA signal and activates the JA signal transduction pathway, thereby increasing enzymatic activities of PPO and POD as well as TrypPI production, which lead to increased rice resistance against chewing insects (Fig. 8).

## Materials and Methods

### Generation of transgenic plants

In the present study, we silenced *OsCOI1* (accession: AY168645) in rice isolated by Hu et al. [49], encoding a putative protein containing an F-box motif and 16 leucine-rich repeats (LRRs). To construct the *OsCOI1* RNAi vector, the 617 bp cDNA fragment of *OsCOI1* was amplified by RT-PCR with primers 5′- gcaggatccgctcaagctcgacaagtgca-3′ and 5′-gctaagcttcaattcggagtcttcgtagc-3′ derived from conserved LRR cDNA regions of *OsCOI1* (Fig. S1). Restriction sites *Bam*HI and *Hind*III were incorporated into the primers, respectively. PCR conditions were 1 min initial denaturation at 94°C, followed by 30 cycles of denaturation for 45 s at 94°C, annealing for 45 s at 52°C and ectension for 8 min at 72°C. Vector pRNAi.5 (Fig. S2, kindly provided by Professor Yao-Guang Liu, College of Life Sciences, South China Agricultural University) was digested by *Bam*HI and *Hind*III enzymes, and the *OsCOI1* fragment was then inserted into *Bam*HI and *Hind*III restriction sites. Both PCR with the specific primers and restriction enzyme digestion verified that the fragment had been correctly inserted into the vector. This first round-ligated vector was then used as the template to amplify a second sequence with two unique restriction sites in both ends (RNAi-*Mlu*I: 5′-caccctgacgcgtggtgttacttctgaagagg-3′; RNAi-*Pst*I: 5′-actagaactgcagcctcagatctaccatggtcg-3′). The second sequence was subsequently cloned between *Pst*I and *Mlu*I, resulting in an opposite orientation in contrast to the first sequence. Restriction digestion showed that the second target fragment had been correctly inserted into the vector. Finally, the DNA sequencing further confirmed the correct orientations sequences 100% identical to that reported in GeneBank (accession: AY168645) (Fig. S3 A and B).

Rice (*Oryza sativa* L. cv. Shishoubaimao) was used for transformation. The construct containing the invert *OsCOI1* sequence repeats driven by the 35S promoter was transferred into rice callus according to an *Agrobacterium* (strain EHA105)-mediated transformation procedure [64]. Calluses were co-cultured for 2 d, and were then screened twice for hygromycin resistance at 40 mg L^−1^, each for 20 d. The selected resistant calluses were put on pre-redifferentiated medium for 15 d and then transferred to redifferentiation medium until the callus produced shoots, and the shoots rooted by transfer to rooting medium. The plantlets were transplanted to soil. Twenty days later, leaves of different T_0_ lines were harvested for analyses of the copy number of *OsCOI1* RNAi construct by Southern hybridization, two homozygous T_0_ lines (L1 and L2) were identified, each harboring a single insertion (Fig. S4A). *OsCOI1* transcripts in these T_0_ lines were not induced by JA (Fig. S4B). The seeds harvested from L1 and L2 were germinated and grown in complete Kimura B nutrient solution, then transferred to normal soil conditions to grow until the seeds were harvested. The relative expression of *OsCOI1* in T_1_ lines was analyzed by qRT-PCR (Fig. S4C). The well silenced individuals were used for seed production. T_2_ seeds were used for the functional analyses in this study.

### Southern blot analysis

Genomic DNA was extracted from the leaves using a cetyl trimethyl ammonium bromide procedure [65]. DNA was digested using *Hind*III restriction enzymes, separated on a 0.8% w/v agarose gel, and transferred to a nylon membrane (Hybond -N^+^, Amersham, United Kingdom). To determine the copy number of *OsCOI1* RNAi construct in transgenic plants, a PCR fragment of the hygromycin phosphotransferase gene amplified by gene-specific primers Hpt-F (5′- tccggagcctccgctcgaagtag-3′) and Hpt-R (5′-ctgaactcaccgcgacgtctgtc-3′) was used as a probe for detection in Southern hybridization. α-^32^P dCTP was used to label the probe using the manufacturer's protocol for the TakaRa random primer labeling kit (TAKARA, http://www.takara-bio.co.jp). Hybridization conditions were as follows: pre-hybridization at 65°C with hybridization buffer (0.25 M NaHPO_4_, pH 7.2, 7% SDS, 1 mM EDTA, 1% BSA) for 60 min, hybridization at 65°C for 15 h, and washing with 2× SSC and 0.1% SDS twice (30 min for each), and then washing with new 2× SSC and 0.1% SDS for 15 min. After autoradiography on a storage phosphor screen, images were scanned using a FX scanner (BIO-RAD).

### Plant growth

Rice seeds of WT and *OsCOI1* RNAi lines were surface-sterilized with 10% H_2_O_2_ and rinsed three times with sterile distilled water. The seeds were presoaked in sterile distilled water for 1 d, pre-germinated for 3 d, and grown in plastic buckets in a greenhouse for 20 d. Seedlings were then transplanted to small plastic pots (diameter 10 cm, height 12 cm), and each pot contained one plant. The soil for plant growth was obtained from the rice fields on the campus of South China Agricultural University in Guangzhou, China. Plants were watered daily, and each pot was supplied with 20 ml of nutrient solution (urea, 1 g L^−1^) every week. All plants were grown in a greenhouse at 28±2°C, with a 12 h light phase and 80% relative humidity. Plants were used for experiments 25–30 days after transplanting.

### Insects

BPH and LF larvae were originally obtained from rice fields of Dafeng Base of Guangdong Academy of Agricultural Sciences, Guangzhou, China, and maintained on WT plants in a climate-controlled room (26±2°C, 80% relative humidity, and 12 h light phase). The BPH nymphs of the third generation and third instars of LF were used for bioassays and feeding treatments.

### Plant treatments

Two third instar LF larvae that had been starved for 2 h were placed on leaves at node 3 and 4 of each individual plant (the youngest fully expanded leaf was defined as leaf node 1). Non-infested control plants were not manipulated. For BPH treatment, each plant was individually infested by 15–20 gravid BPH contained in two parafilm bags (6×5 cm), each bag was then fixed to upper and lower positions on the stems. Two empty bags were fixed to control (non-infested) plants.

Plants (one plant per pot) were individually sprayed with 1 ml of MeJA (1 mM) with 0.01% Tween 20 for 48 h. Control plants were sprayed with 1 ml of the buffer with 0.01% Tween 20.

For LF and MeJA treatments, node 4 of leaves was harvested for analyses of gene expression, TrypPI content, enzyme activities and JA level analysis. In the BPH treatment, leaf sheathes were harvested for analysis. There were six biological replicates for each treatment.

### Quantitative real-time PCR analysis

Differential expression of selected genes was verified by quantitative real-time PCR (qRT-PCR) using the RNA samples isolated from rice tissues obtained from different treatments. The actin gene was used as a reference gene. Total RNA from rice leaves was extracted according to the method as described by Kiefer [66] including a DNase (Promega, Madison, USA) treatment. First strand cDNA was synthesized from 1 µg of total RNA using ImProm-II™ Reverse transcription system (Promega, Madison, USA) according to the manufacturer's instructions. The primers for target gene *OsCOI1* were designed by Primer 5.0 software (Applied Biosystems, http://fokker.wi.mit.edu/primer3/input.htm). We used the following primers: *OsCOI1* sense, 5^′^-ttgccgtgaattggagtacatag-3^′^ and antisense 5^′^-^,^gtcaagtagcacaagccgaaag-3^′^ ; *OsActin* (Internal standard, accession: X15865) sense, 5^′^- ctgacggagcgtggttac-3^′^ and antisense 5^′^-ggaaggcgggaagaggac -3^′^ . Real-time PCR reactions were carried out with 0.2 µl (0.15 µM) of each specific primer, 1 µl of cDNA, 12.5 µl of the SYBR green master mix (Quanti Tech SYBR Green kit, Qiagen, Gmbh Hilden, Germany) and the final volume was adjusted to 25 µl with RNase-free water. Reactions were performed on a DNA Engine Opticon 2 Continuous Fluorescence Detection System (MJ Research Inc., Waltham, MA). The program used for real-time PCR was 3 min initial denaturation at 95°C, followed by 35 cycles of denaturation for 20 s at 95°C, annealing for 20 s at 58°C for all genes and extension for 20 s at 72°C. The fluorescence signal was measured immediately after incubation for 2 s at 75°C following the extension step, which eliminates possible primer dimer detection. At the end of the cycles, melting temperatures of the PCR products was determined between 65°C and 95°C. The specificity of amplicons was verified by melting curve analysis and agarose gel electrophoresis. Three independent biological replicates for each treatment were used for qRT-PCR analyses. Relative expression of target gene was calculated by Double-stand Curves method.

### Bioassays

#### LF performance measurement

Thirty *OsCOI1* RNAi plants were divided into five groups according to the transcripts level of *OsCOI1*. Six RNAi plants with approximately equal expression level of *OsCOI1* placed into each group. As a result, 30 RNAi plants were divided into five groups. Additionally, fifteen WT plants served as control. Two second-instar LF larvae were placed individually on the node 3 and 4 leaves of WT and RNAi plants. So there were 6 plant replicates with 12 LF larvae for each RNAi group, and 15 plant replicates with 30 LF larvae for WT. Larval weight was measured to an accuracy of 0.1 mg three days after the larvae were placed on plants, and the increased percentage of larval weight on each plant was calculated.

#### BPH performance measurement

To measure BPH feeding on WT and RNAi lines, three newly emerging macropterous female BPH adults, starved for 2 h, were placed into a small parafilm bag (6×5 cm), which was then fixed on the stems of plants, with each plant receiving three females. The amount of honeydew excreted by three female adult was weighed (to an accuracy of 0.1 mg) 24 h after the start of the experiment. The experiment was replicated 10 times.

The survival rates of BPH nymphs on WT and RNAi lines were also determined. Pots with one plant were individually covered with plastic cages (diameter 10 cm, height 30 cm) into which fifteen newly hatched BPH nymphs were released. The number of surviving BPH nymphs on each plant was recorded 5 d after insect infection. The experiment was repeated 10 times.

### Enzyme Assays

Samples (0.1 g) harvested from rice plants subjected to different treatments were ground to fine powder in liquid nitrogen, and homogenized in 2.0 ml of ice cold 0.05 M phosphate buffer (pH 7.2 for POD, pH 7.8 for PPO) containing 1% (w/v) polyvinylpyrrolidone. The homogenate was centrifuged at 12000 *g* for 15 min at 4°C. The supernatant was collected and used for assay of POD and PPO activities using spectrophotometer. There were six biological replicates in each treatment.

POD activity was determined as described by Kraus and Fletcher [67]. PPO activity was assayed with 0.05 M catechol as a substrate by a spectrophotometric procedure [68].

LOX activity was measured as conjugated diene formation [69]. Leaf samples (0.1 g) were ground in liquid nitrogen and extracted with 1 ml of ice-cold 0.5 M Tris-HCl buffer (pH 7.6) and centrifuged at 12000 *g* for 15 min at 4°C. The supernatant was kept at 4°C until used. The substrate contained 1.6 mM linoleic acid and 0.5% (v/v) Tween 20 in 0.1 M phosphate buffer (pH 7.6). The reaction was initiated by the addition of 0.2 ml of crude extract in 4.8 ml of the substrate. Diene formation was followed as increase of absorbance at 234 nm.

### TrypPI analyses

TrypPI activity was measured using a colorimetric assay which uses the protein chromophore azocasein as a substrate [70]. In our modified assay, leaf tissue (0.1 g) was ground in 0.2 M Tris-HCl buffer (pH 8.0) with 0.1% Tween 20, samples were centrifuged at 12000 *g* for 20 min at 4°C and the supernatant was collected. Each reaction contained 200 µl plant extract. 500 µl of 0.1 µg/µl trypsin was added to each reaction, mixed and left at room temperature for 10 min. 100 µl of 25 mg/ml azocasein was added, the reaction mixed and incubated at 37°C for 40 min. Samples were centrifuged at 12000 *g* for 10 min, then 200 µl of supernatant was mixed with 200 µl of 0.5 M NaOH and the absorbance measured at 450 nm. The amount of protease inhibitor as nmol in each sample was calculated based on a standard curve, and results were expressed as nm protease inhibitor per mg protein, with protein determined by the Bradford assay [71] using BSA as standard.

### JA and SA analyses

Plants (one per pot) were randomly assigned to LF and non-infested treatments. The leaves were harvested at 0, 1.5, 3 and 8 h after treatment. Leaf samples were immediately frozen in liquid nitrogen and stored at −80°C. For each time point and treatment, six plants were sampled. JA and SA content was measured by GC analyses using external JA and SA standards (Sigma-Aldrich, St. Louis, MO, USA) as described by Song *et al.* [72]. Samples were extracted by mixture of acetone and citric acid (50 mmol L^−1^) (v/v = 7/3), and ethyl acetate. Then the supernatant was dried by N_2_ and subsequently methylated with trimethylsilyldiazomethane. The volatilized compounds were collected by using headspace-solid phase microextraction (HS-SPME) on Tenax adsorbents' and eluted with n-hexane. Eluted samples were analyzed by using GC with hydrogen ion flame detector (FID). The temperature gradient was increased from 60°C (1 min) to 250°C in a rate of 15°C/min and held on 3 min at 250°C. The final chromatographic peaks of JA and SA in the samples were identical to the authentic compounds (Fig. S6). 25 µl 80 µg/ml JA and 125 µl 160 µg/ml SA were mixed, and after the step of extraction and methylation with trimethylsilyldiazomethane as samples, 100 µl n-hexane was used to elute the MeJA and MeSA collected in Tenax by HS-SPME, the mixed MeJA (20 µg/ml) and MeSA (200 µg/ml) were diluted into several concentration to be used as stands to quantify JA and SA levels of samples. In addition, mixed standard MeJA (18 µg/ml) and MeSA (40 µg/ml) (Sigma-Aldrich, St. Louis, MO, USA) were used to confirm the recovery rate of JA and SA. The method resulted in a high level of recovery, reproducibility, and linearity in the quantification of JA and SA (Fig. S7; Table S1).

### Statistical analysis

SPSS 14.0 (SPSS, Chicago, IL, USA) package for Windows was used for statistical analysis. Differential gene expression, enzymatic activities, and TrypPI level of LF- or BPH-infested and their respective non-infested WT control plants were determined using Student's t-test. Differential *OsCOI1* expression caused by LF, MeJA or BPH treatment at each time point as compared to control plants respectively was determined using Student's t-test. For LF performance on exogenous MeJA-treated and untreated control WT plants and BPH performance on WT and RNAi lines, Student's t-tests were used. Differences in LF performance on WT and RNAi lines, LF-induced gene expression, enzymatic activities, and TrypPI level, JA and SA levels at each time point on WT and RNAi lines were evaluated by Tukey post-hoc test one-way ANOVA at *P* = 0.05.

## Supporting Information

Figure S1 **Nucleotide sequence and amino acid sequence of targeted** ***OsCOI1*** **gene (accession: AY168645) and RNAi target region of the hairpin-forming RNAi transgene cassette used in the present study.** F-box motif is indicated by double underline. Leucine rice repeats (LRRs) were marked by single underline. RNAi target region is shown in red, the primers (OsCOI1 5′ and OsCOI1 3′) derived from conserved domains in LRRs region are indicated below the nucleotide sequence.
(TIF)

Figure S2 **Rice transformation vector pRNAi-COI1 with** ***HPT*** **as plant selectable marker gene.**
(TIF)

Figure S3 (A) cDNA sequence of amplified *OsCOI1* fragment. (B) Identity analyses of amplified *OsCOI1* fragment.
(TIF)

Figure S4 (A) DNA gel-blot analysis of two T_0_ *OsCOI1* RNAi lines and one WT line. (B) RT-PCR analysis of transcriptional expression of *OsCOI1* from the T_0_ *OsCOI1* RNAi lines and WT plants (C: Control, no manipulation; T: treated with 100 nmol L^−1^ JA). (C) Relative expression of *OsCOI1* in WT and T_1_ RNAi lines.
(TIF)

Figure S5 **Growth phenotype of** ***OsCOI1*** **RNAi lines and WT rice plants.**
(TIF)

Figure S6 **Profiles of GC chromatography of authentic MeJA, MeSA and JA derived MeJA and SA derived MeSA in rice leaves.**
(TIF)

Figure S7 **Recovery rates of jasmonic and salicylic acids in the GC analysis.**
(TIF)

Table S1 **Retention time, linear regression equation and limit of detection of JA and SA detected by GC-FID.**
(DOC)
