# Supplementary figures and images for: Silencing COI1 in Rice Increases Susceptibility to Chewing Insects and Impairs Inducible Defense
Source: PLoS One. 2012 Apr 27;7(4):e36214. doi: 10.1371/journal.pone.0036214 (PMC3338713; doi:10.1371/journal.pone.0036214)

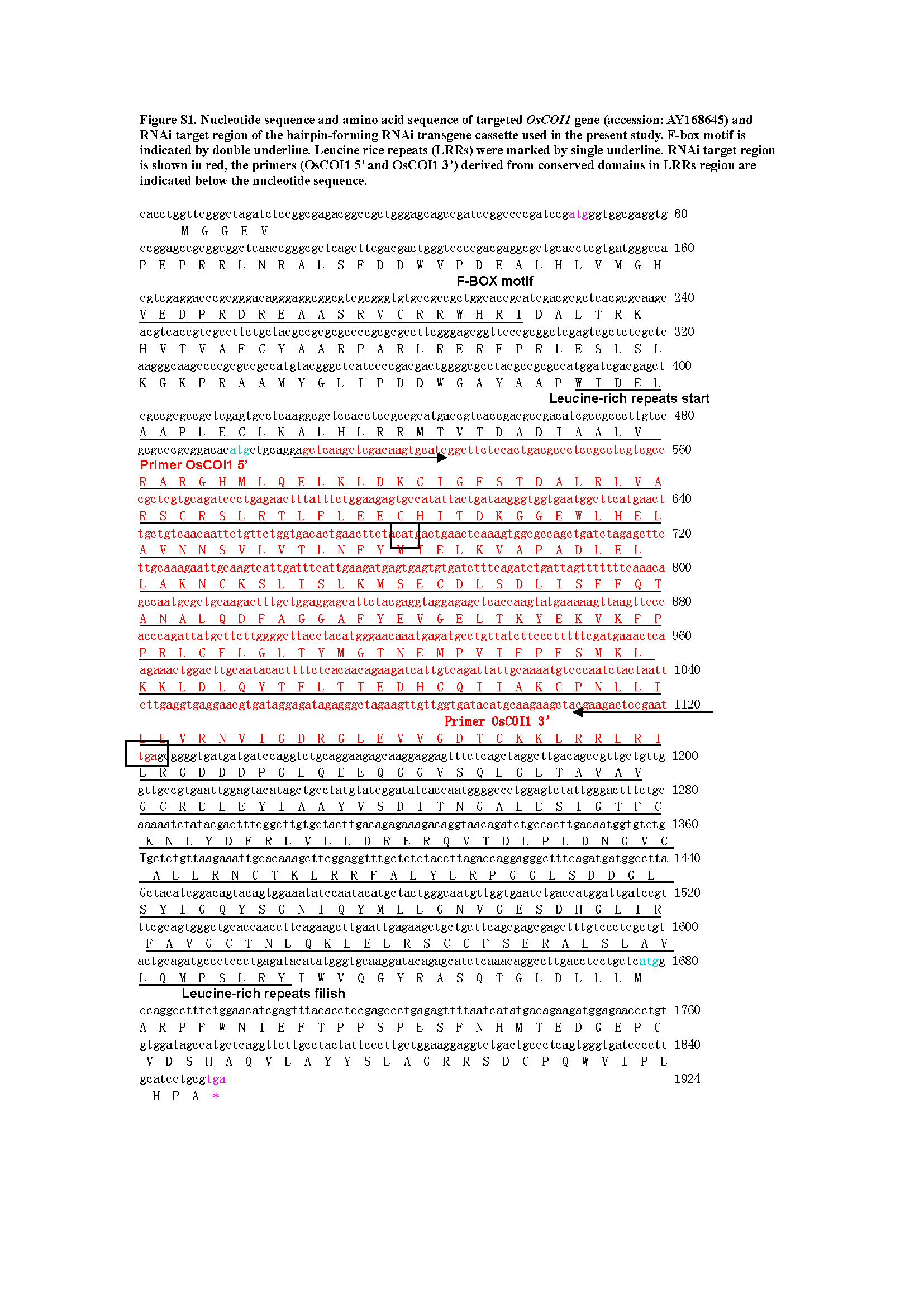

Supplement: Figure S1 — Nucleotide sequence and amino acid sequence of targeted OsCOI1 gene (accession: AY168645) and RNAi target region of the hairpin-forming RNAi transgene cassette used in the present study. F-box motif is indicated by double underline. Leucine rice repeats (LRRs) were marked by single underline. RNAi target region is shown in red, the primers (OsCOI1 5′ and OsCOI1 3′) derived from conserved domains in LRRs region are indicated below the nucleotide sequence. (TIF) [file pone.0036214.s001.tif]

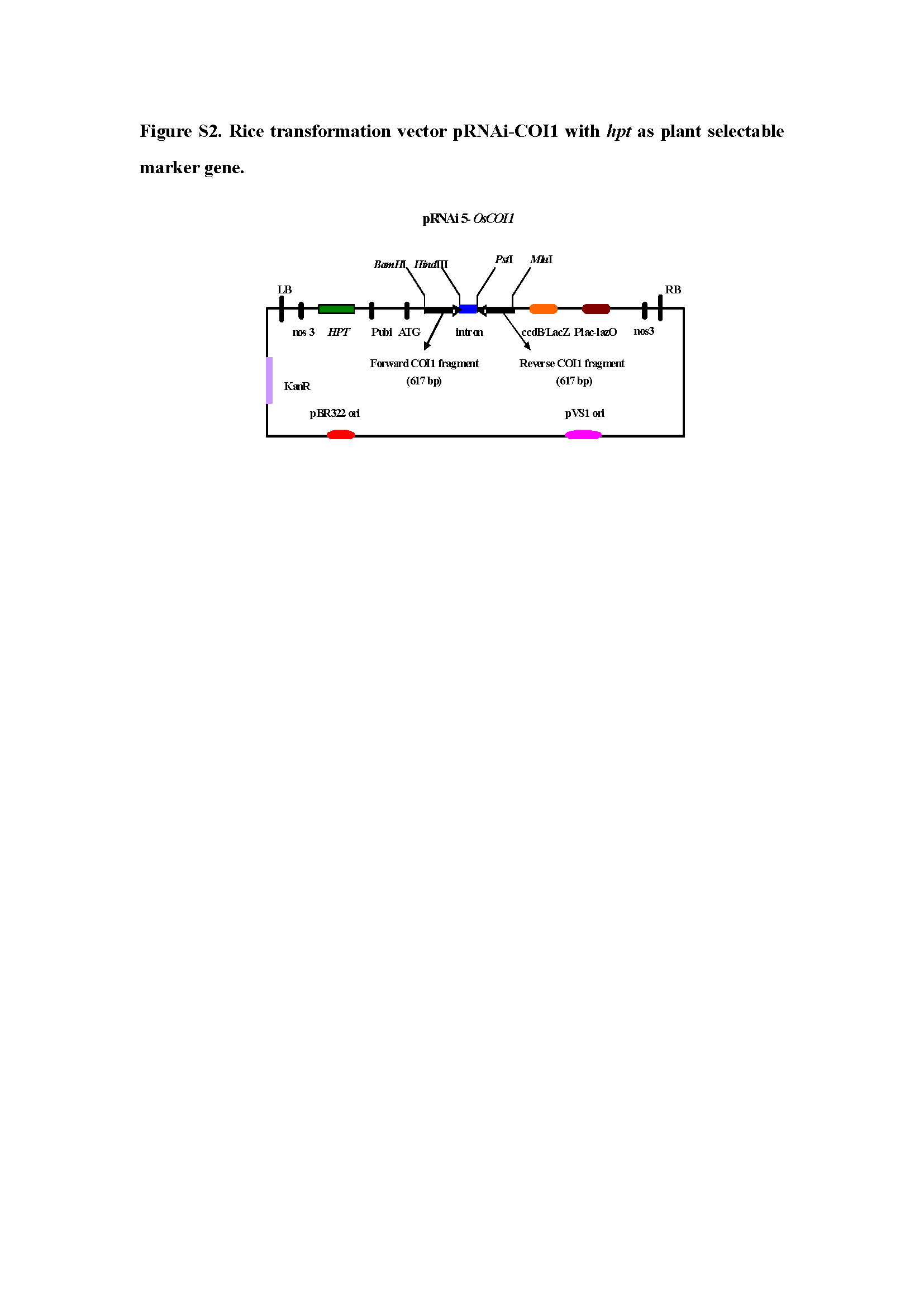

Supplement: Figure S2 — Rice transformation vector pRNAi-COI1 with HPT as plant selectable marker gene. (TIF) [file pone.0036214.s002.tif]

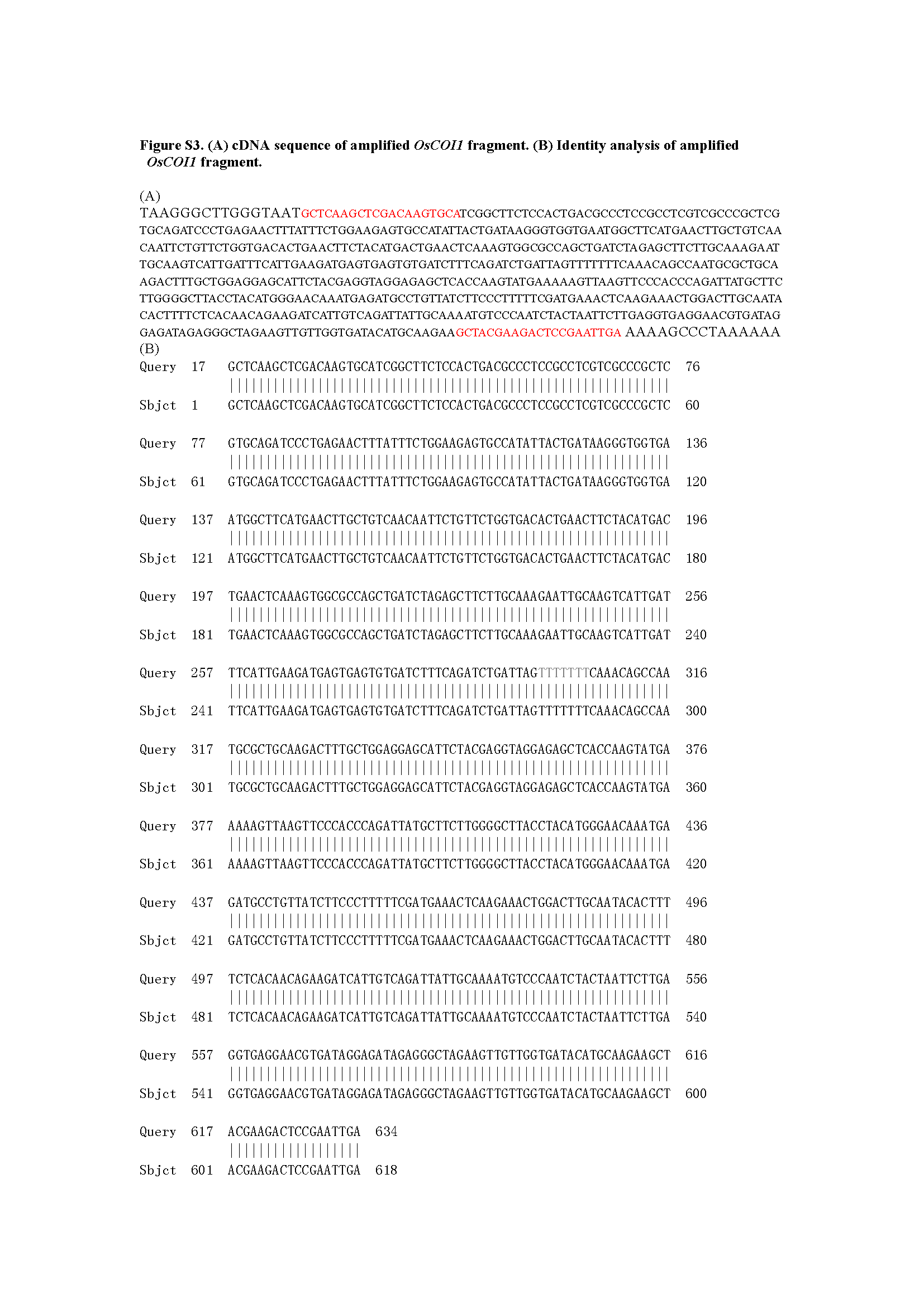

Supplement: Figure S3 — (A) cDNA sequence of amplified OsCOI1 fragment. (B) Identity analyses of amplified OsCOI1 fragment. (TIF) [file pone.0036214.s003.tif]

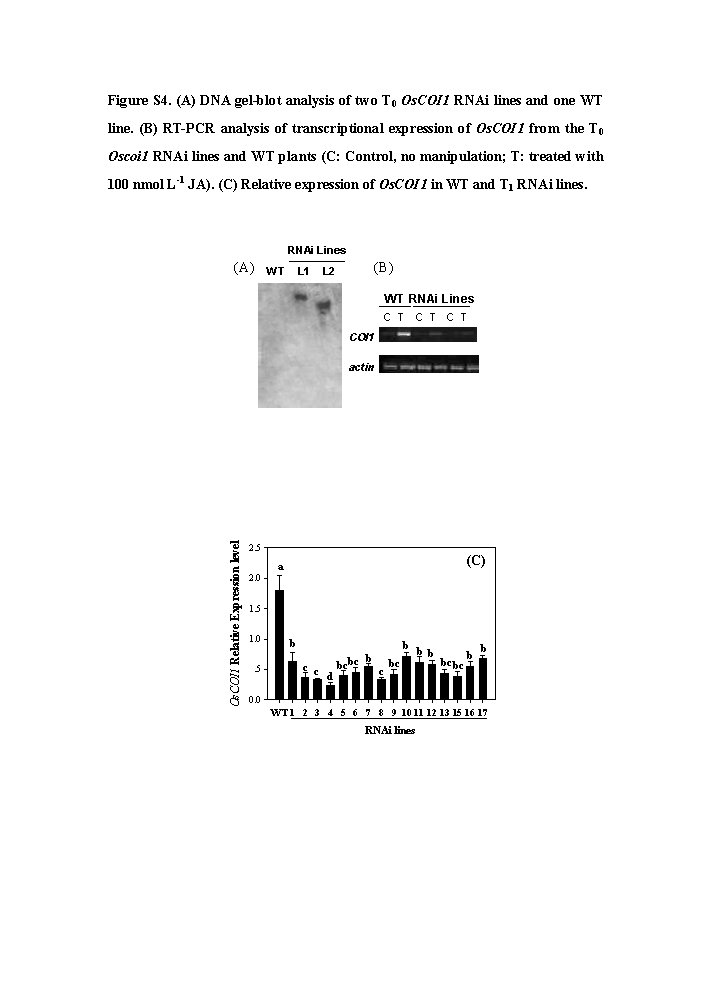

Supplement: Figure S4 — (A) DNA gel-blot analysis of two T0 OsCOI1 RNAi lines and one WT line. (B) RT-PCR analysis of transcriptional expression of OsCOI1 from the T0 OsCOI1 RNAi lines and WT plants (C: Control, no manipulation; T: treated with 100 nmol L−1 JA). (C) Relative expression of OsCOI1 in WT and T1 RNAi lines. (TIF) [file pone.0036214.s004.tif]

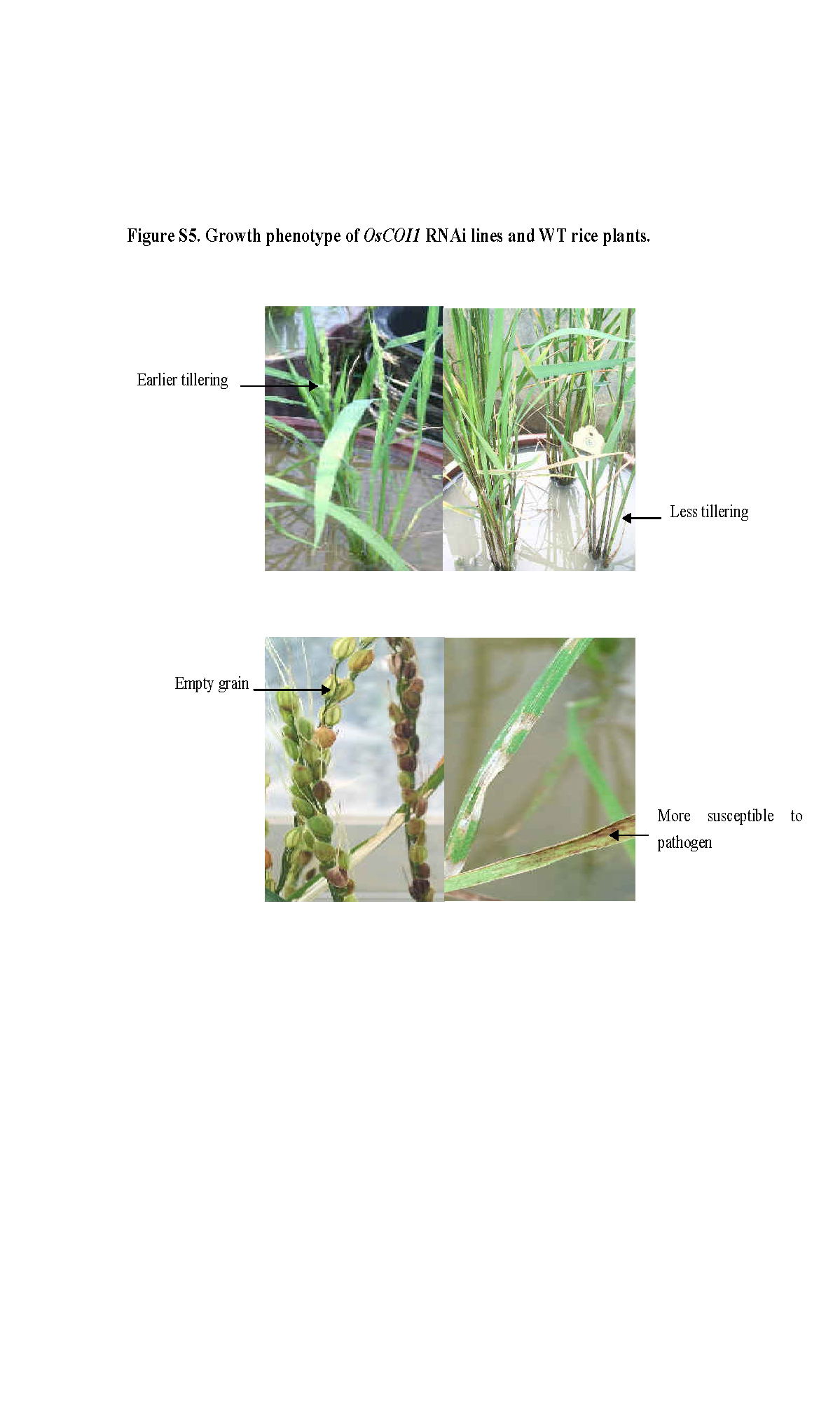

Supplement: Figure S5 — Growth phenotype of OsCOI1 RNAi lines and WT rice plants. (TIF) [file pone.0036214.s005.tif]

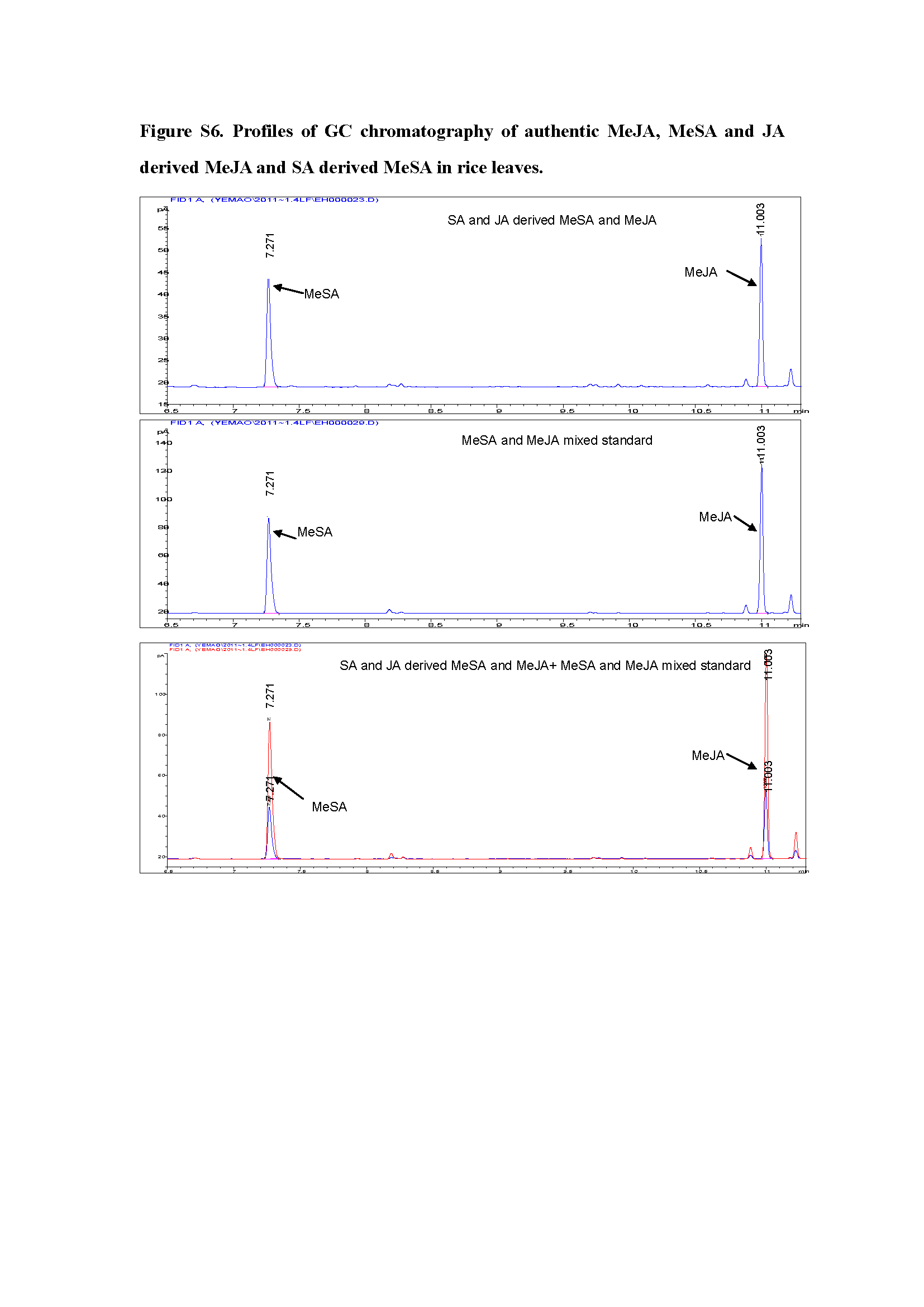

Supplement: Figure S6 — Profiles of GC chromatography of authentic MeJA, MeSA and JA derived MeJA and SA derived MeSA in rice leaves. (TIF) [file pone.0036214.s006.tif]

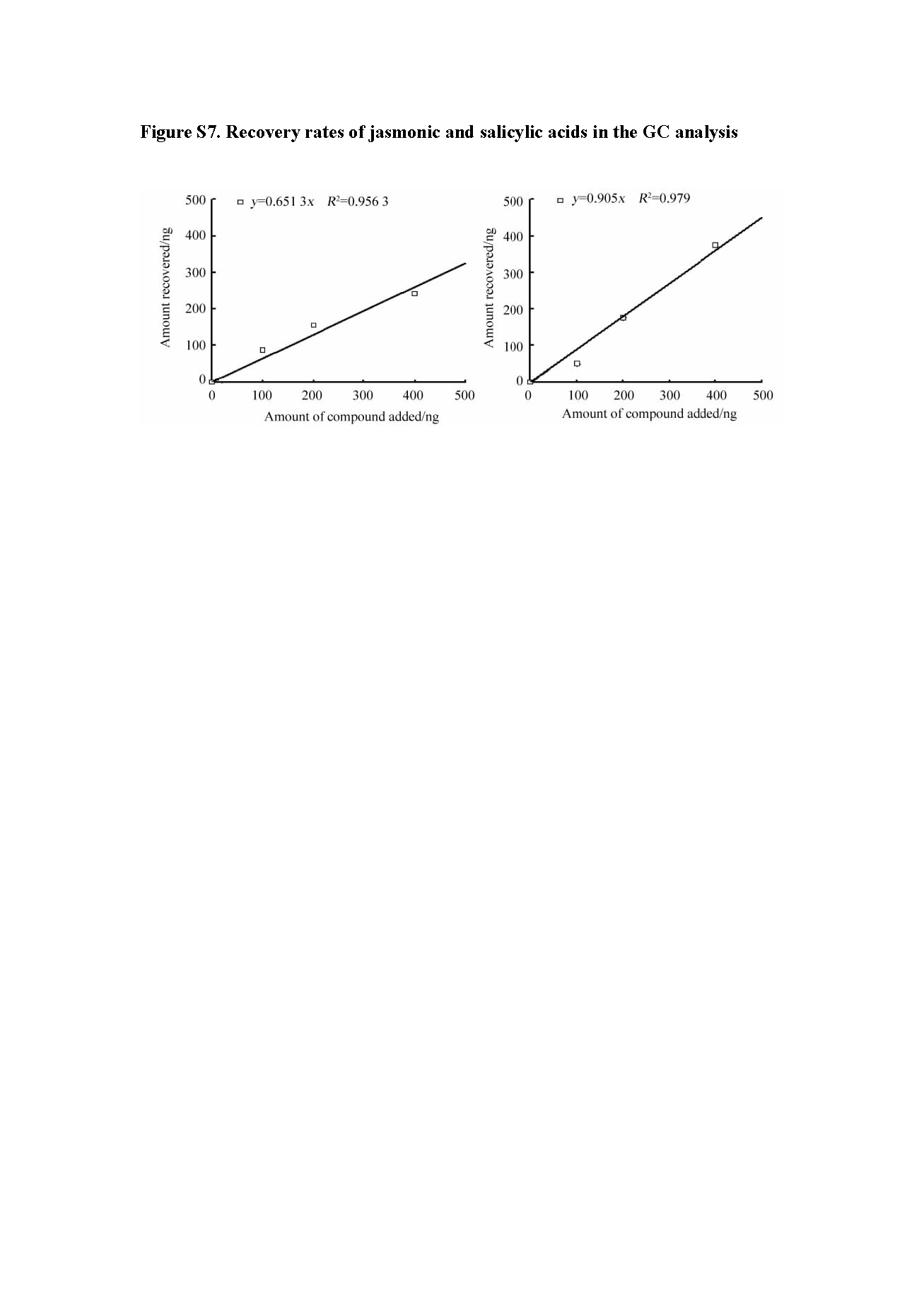

Supplement: Figure S7 — Recovery rates of jasmonic and salicylic acids in the GC analysis. (TIF) [file pone.0036214.s007.tif]
