# Supplementary material for: Silencing COI1 in Rice Increases Susceptibility to Chewing Insects and Impairs Inducible Defense
Source: PLoS One. 2012 Apr 27;7(4):e36214. doi: 10.1371/journal.pone.0036214 (PMC3338713; doi:10.1371/journal.pone.0036214)
Supplement: Table S1 — Retention time, linear regression equation and limit of detection of JA and SA detected by GC-FID. (DOC) [file pone.0036214.s008.doc]

**Table S1 Retention time, linear regression equation and limit of detection of JA and SA detected by GC-FID**

| Compound | Retention time | Linear regression | Linearity range | Coefficient | Detection limit |
| --- | --- | --- | --- | --- | --- |
|  | (min) |  | (ng μl^-1^) |  | (ng μl^-1^) |
| Salicylic acid | 7.271 | Area=30.1200121×Amt-25.753772 | 0.5-40.0 | 0.99921 | 0.100030 |
| Jasmonic acid | 11.003 | Area=37.3437531×Amt-5.0685045 | 0.225-18.000 | 0.99986 | 0.080680 |
